# Supplementary material for: Toward a methodology for evaluating DNA variants in nuclear families
Source: PLoS One. 2021 Oct 8;16(10):e0258375. doi: 10.1371/journal.pone.0258375 (PMC8500447; doi:10.1371/journal.pone.0258375)
Supplement: Attachment — Submitted filename: PedFam_response_to_reviewers_PLOS.pdf [file pone.0258375.s005.pdf]

DEPARTMENT OF BIOLOGY

Dear editors:

Thank you for taking the time to review our manuscript entitled, "Toward a methodology for evaluating DNA variants in nuclear families". We appreciate the reviewer's helpful comments. We have addressed these comments below in a point-by-point response. These changes have helped to improve the manuscript.

We have also made some formatting changes requested by the journal and made some additional wording changes for clarification and consistency reasons. We have included a "tracked changes" version of the manuscript.

Please let me know if I can address any questions you may have.

Warm regards,

Stephen R. Piccolo, PhD
Associate Professor
Department of Biology
Brigham Young University
(801) 422-7116
Stephen_Piccolo@byu.edu

The editor's and reviewers' comments are in this color. Our responses are in black text.

Journal requirements:

When submitting your revision, we need you to address these additional requirements.

1. Please ensure that your manuscript meets PLOS ONE's style requirements, including those for file naming. The PLOS ONE style templates can be found at

https://journals.plos.org/plosone/s/file?id=wjVg/PLOSOne_formatting_sample_main_body.pdf and

https://journals.plos.org/plosone/s/file?id=ba62/PLOSOne_formatting_sample_title_authors_affiliations.pdf

We have updated our manuscript to address these requirements. Please let us know if anything else

needs to be changed with the formatting.

2. Thank you for stating the following in the Acknowledgments Section of your manuscript:
"Brigham Young University provided startup funds to SRP and a graduate assistantship to DBM."

We note that you have provided funding information that is not currently declared in your Funding Statement. However, funding information should not appear in the Acknowledgments section or other areas of your manuscript. We will only publish funding information present in the Funding Statement section of the online submission form.

Please remove any funding-related text from the manuscript and let us know how you would like to update your Funding Statement. Currently, your Funding Statement reads as follows:

"The author(s) received no specific funding for this work"

Please include your amended statements within your cover letter; we will change the online submission form on your behalf.

Thank you for letting us know. We have removed our previous statement from the manuscript. Please change our Funding Statement to: "The College of Life Sciences at Brigham Young University provided faculty startup funds to SRP. The Department of Biology at Brigham Young University provided a graduate assistantship to DBM."

3. Please review your reference list to ensure that it is complete and correct. If you have cited papers that have been retracted, please include the rationale for doing so in the manuscript text, or remove these references and replace them with relevant current references. Any changes to the reference list should be mentioned in the rebuttal letter that accompanies your revised manuscript. If you need to cite a retracted article, indicate the article's retracted status in the References list and also include a citation and full reference for the retraction notice.

We are unaware of any references that have been retracted. We went through and checked these in PubMed and did not find any that had been retracted. Please let us know if you are aware of any that we missed.

Reviewer #1:

The manuscript entitled "Toward a methodology for evaluating DNA variants in nuclear families" is a rather interesting approach to evaluate pediatric cancer risk of children carrying rare variants. Data are exhaustive and the manuscript deserves publication after minor revision just to increase clarity for a non expert reader. Manuscript is well written and methods well explained; however, I recommend some change in the figure legends.

We thank the reviewer for taking time to review the manuscript and for these encouraging comments.

Figure 1 needs more detailed legends. Question marks mean that children have cancer or not? or not sure? Figure two is fine.

We have added clarifying details to the legend for Figure 1, as shown below:

"Each diagram in this figure represents one family from our study. In each diagram, shapes at the top represent parents; shapes at the bottom represent children. Numbers below children are sample identifiers. These identifiers do not necessarily correspond to birth order or any other factor. One child in each family had been diagnosed with pediatric cancer, but the disease status of all children was unknown to the primary author until after disease-history predictions were made. This unknown status is represented using question marks."

From figure 3, I understand that sample ID 29 (from family 1) carries 16 SNPs, 2 indels and 2 SV; totally, 20 potentially damaging variants, right?

That is correct. We have added more details to the figure legend to help clarify, as shown below:

"This figure shows the number of potentially damaging variants for each variant type (simple heterozygous, homozygous alternate, and compound heterozygous) and category (SNP, indel, and SV) in each sample. Potentially damaging variants met specific MAF, CADD and impact severity thresholds (see Methods)."

In table 1, family 1, 1 child carries 12 damaging variants: are these the same data as reported in figure 3? Please explain.

We apologize for the confusion. Although Table 1 and Figure 3 are complementary, they provide different types of information. Figure 3 illustrates variants that were present in each individual in each family, whereas Table 1 summarizes the overlap in variants among siblings in a given family. Both types of information are important to communicate. We have rewritten the legend for Table 1 to clarify what is shown in that table:

"Table 1. An overview of variants in common among children within each nuclear family. We excluded variants that were present in more than two children in a given nuclear family. This table shows the number of variants in common among a specified number of siblings. For example, in Family 1, 12 variants (in 10 genes) were unique to a single child; 5 variants (in 5 genes) were shared between two children. These numbers reflect the number of "potentially damaging" variants—including simple-heterozygous, compound-heterozygous, and homozygous-alternate variants—that met specific MAF, CADD and impact-severity thresholds (see Methods)."

Supplementary tables are exhaustive, discussion is fine; however, the authors should point out more

convincingly, the importance of the study.

We thank the reviewer for this recommendation. We have added a paragraph at the beginning of the Discussion section that describes additional ways that our study is unique and important:

"This study contributes a novel methodology for estimating the translational importance of DNA variants in a given family. Unlike other designs that use trios or quads or that focus primarily on affected family members, we collect data mostly from unaffected individuals, including parents and siblings. Despite the inherent small sample size of comparing individuals within a nuclear family, family members are often exposed to environmental conditions, cultural practices, and dietary habits that are more similar to each other than to the general population, thus potentially reducing confounding factors that could bias an analysis. Although we were not able to demonstrate that our predictions were more accurate than expected by random chance, we have introduced a methodology for identifying candidate variants that can be expanded or refined. Furthermore, we have shared a unique dataset with the community; we are unaware of other WGS datasets from nuclear families with as many as seven children."

Later in the Discussion section, we highlight additional factors that make this study unique and important. For example, we explain how our study differs from population-based studies, how we combined quantitative and qualitative methods, the ability to include a wide range of variants (including compound-heterozygous and de novo variants that are not profiled in most genetic studies), our methodology for combining multiple lines of evidence from diverse sources, and insights we gained about potential biases. Finally, even if this manuscript reports some negative results, we are confident that it meets all of the PLOS Publishing Criteria.

Thanks again!
